# Prx1-Expressing Progenitor Primary Cilia Mediate Bone Formation in response to Mechanical Loading in Mice

**DOI:** 10.1155/2019/3094154

**Published:** 2019-11-11

**Authors:** Emily R. Moore, Julia C. Chen, Christopher R. Jacobs

**Affiliations:** Department of Biomedical Engineering, Columbia University, 500 W 120th Street New York, NY 10027, USA

## Abstract

Increases in mechanical loading can enhance the addition of new bone, altering geometry and density such that bones better withstand higher forces. Bone-forming osteoblasts have long been thought to originate from progenitors, but the exact source is yet to be identified. Previous studies indicate osteogenic precursors arise from Prx1-expressing progenitors during embryonic development and adult fracture repair. However, it is unknown whether this cell population is also a source for mechanically induced active osteoblasts. We first identified that Prx1 is expressed in skeletally mature mouse periosteum, a thin tissue covering the surface of the bone that is rich in osteoprogenitors. We then traced Prx1 progenitor lineage using a transgenic mouse model carrying both a Prx1-driven tamoxifen-inducible Cre and a ROSA-driven lacZ reporter gene. Cells that expressed Prx1 when compressive axial loading was applied were detected within the cortical bone days after stimulation, indicating osteocytes are of Prx1-expressing cell origin. In addition, we evaluated how these cells sense and respond to physical stimulation *in vivo* by disrupting their primary cilia, which are antenna-like sensory organelles known to enhance mechanical and chemical signaling kinetics. Although Prx1-driven primary cilium disruption did not affect osteoblast recruitment to the bone surface, the relative mineral apposition and bone formation rates were decreased by 53% and 34%, respectively. Thus, this cell population contributes to load-induced bone formation, and primary cilia are needed for a complete response. Interestingly, Prx1-expressing progenitors are easily extracted from periosteum and are perhaps an attractive alternative to marrow stem cells for bone tissue regeneration strategies.

## 1. Introduction

One way the skeleton structurally adapts to its mechanical environment is by stimulating the addition of new bone and subsequently altering its geometry and density to better withstand higher forces. Bone formation in response to mechanical loading involves multiple cell types and requires a sequence of events to occur. Specifically, mechanosensitive osteocytes sense physical loading and secrete paracrine factors that recruit cells to the bone surface [1]. These cells eventually transform into matrix-producing osteoblasts and, potentially, embedded osteocytes. With innovative regenerative bone therapies rapidly emerging, it is more important than ever to finally determine the origin of cells recruited to the bone surface. Bone-forming cells have long been thought to originate from progenitors, so approaches were developed to extract osteoblast precursors from bone marrow. However, these procedures are very invasive, and the acquired progenitors require further treatment in order to encourage proper differentiation. An appealing alternative is to harvest periosteum, which surrounds bones and is rich in progenitor cells known to preferentially differentiate towards the osteogenic lineage [2–4].

Previous *in vitro* studies demonstrate that immortalized murine and primary human mesenchymal stem cells directly sense physical stimulation, which enhances differentiation towards the osteogenic lineage [5, 6]. In addition, mechanical forces on the periosteum are known to enhance osteogenic lineage commitment *in vivo* and *in vitro*. Henderson et al. observed the localization of cells with osteogenic gene expression to areas of tension on the outer edges of embryonic mouse rudiment tissues [7], and Kanno et al. found osteogenic markers were upregulated when mechanical strain was applied to human periosteal cells [8]. These studies suggest that physical stimulation activates and encourages osteogenic differentiation of progenitors within the periosteum. Although progenitors are clearly receptive to mechanical cues, how they sense and react to physical stimulation *in vivo* remain unknown.

The inner cambium layer of the periosteum contains a wealth of osteogenic precursors, a subset of which express paired-related homeobox 1 (Prx1). During embryonic development, Prx1 expression is rampant in the limb bud and gives rise to many skeletal tissues [4]. Prx1 tracking studies in adult mice have identified recombination in perivascular stromal cells [9, 10], mature osteoblasts [9, 10], osteocytes [9], and adipocytes [11]. These results suggest the Prx1Cre transgene is associated with multipotent mesenchymal progenitors in the appendicular skeleton [9]. We recently determined that Prx1 is highly restricted to the periosteum and perichondrium after birth [12] and are further confined to the periosteum in adulthood [13]. Prx1-expressing cells populate the callus during fracture healing [4, 14], but their presence under normal physiological conditions has yet to be confirmed. Thus, we investigate the role of Prx1-expressing progenitors during mechanical loading in this study.

One potential mechanism by which progenitor cells may become mechanically activated is through the primary cilium. Primary cilia are antenna-like organelles that extend from the cell surface and serve as signaling microdomains. Impairment of primary cilium formation and signaling is known to influence bone development and formation [15–17]. For example, when an intraflagellar retrograde transport protein important for primary cilium function (Kif3a) was deleted in osteoblasts and osteocytes, load-induced bone formation was diminished [18]. Primary cilia are also critical for mechano- and chemosensation in mesenchymal progenitor cells *in vitro* [5, 19]. Interestingly, Prx1-driven deletion of primary cilia in murine embryos alters lineage commitment, resulting in severe defects in endochondral bone formation and, ultimately, death [16]. We recently determined that the osteogenic response to fluid shear is lost when periosteal progenitor primary cilia are disrupted *in vitro* [13]. Despite the implications, whether Prx1-expressing progenitor cell primary cilia mediate the *in vivo* bone formation response to mechanical stimulation has yet to be investigated.

The objectives of this study are twofold. First, we traced Prx1-expressing cells in skeletally mature adults to examine their fate in load-induced bone formation. Second, we measured changes in load-induced bone formation with and without Prx1-expressing progenitor primary cilia to evaluate their role in adult adaptation. Overall, we determined that Prx1-expressing cells become embedded osteocytes in response to physical loading and this mechanism requires the primary cilium.

## 2. Methods

### 2.1. Generation of Prx1CreER-GFP; Kif3a^fl/fl^ and Prx1CreER-GFP; R26R-lacZ Mice

**Figure 1:**
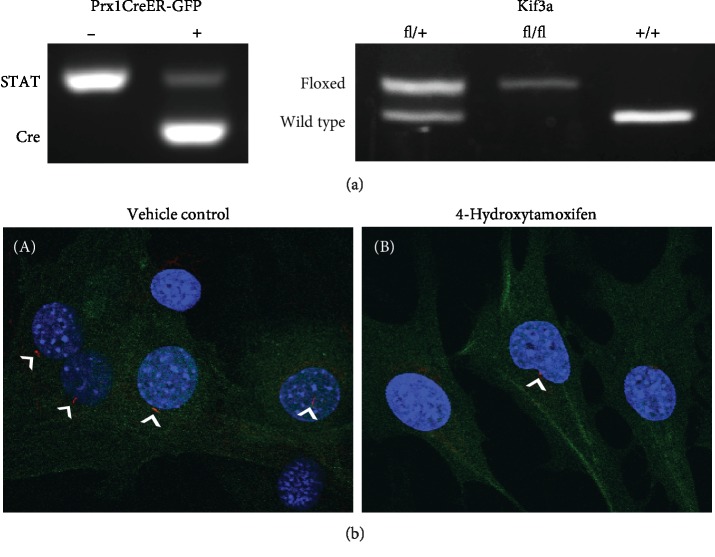
Generation of experimental Prx1CreER-GFP; Kif3a^fl/fl^ and control mice. Genotype was confirmed using PCR to detect the Prx1CreER-GFP transgene and Kif3a wild-type and floxed alleles (a). The signal transducer and activator of transcription (STAT) gene served as a positive control for the PCR reaction. IHC for acetylated *α*-tubulin was performed to visualize primary cilia (b). Cilium (red, white arrows) incidence and length decreased in Prx1CreER-GFP; Kif3a^fl/fl^ primary periosteal cells (green) treated with 4-hydroxytamoxifen (B) compared to controls (A). Nuclei (blue) were stained using DAPI, and micrographs were collected at 100x magnification.

All mice were maintained in the animal facility at Columbia University with protocols approved by the Institutional Animal Care and Use Committee at Columbia University. C57BL/6 mice carrying floxed alleles of Kif3a were recovered from the UC Davis Mutant Mice Regional Resource Center cryoarchive. C57BL/6 mice carrying the Prx1CreER^T2^ transgene were transferred from Case Western University. Mice carrying ROSA26-lacZ (R26R) were obtained from Jackson Laboratories (Bar Harbor, ME) and bred with Prx1CreER-GFP animals to generate the Prx1CreER-GFP; R26R^tg/+^ model for the lineage-tracing experiment. For ulnar loading studies, Kif3a^fl/fl^ and Prx1CreER-GFP animals were crossed to generate experimental Prx1CreER-GFP; Kif3a^fl/fl^ mice (Figure 1(a)). The control group includes multiple genotypes that retain both copies of Kif3a upon tamoxifen administration: (1) Kif3a^fl/fl^, (2) Prx1CreER; Kif3a^+/+^, (3) Prx1CreER; Kif3a^+/+^; R26R^tg/+^, and (4) Kif3a^fl/+^. Genotypes were verified by extracting DNA from tail biopsies and performing PCR analysis [4, 20]. Both female and male mice were used for all experiments. All applicable institutional and/or national guidelines for the care and use of animals were followed.

### 2.2. In Vitro Cilium Disruption and Immunocytochemistry

16-week-old Prx1CreER-GFP; Kif3a^fl/fl^ mice were sacrificed, and the limbs were dissected. The skin, muscle, and connective tissues were removed to expose the periosteum. The periosteum was gently scored with a scalpel, peeled from the bone, cut into 1 mm × 1 mm sections, and placed into culture media (MEM*α*+10% FBS+1% PenStrep, Thermo Fisher Scientific, Waltham, MA) in a fibronectin-coated (Sigma Chemical Co., St. Louis, MO) 35 mm tissue culture dish (Falcon, Corning, NY). Tissue sections were cultured at 37°C for 7–10 days, and the resulting isolated cells were seeded on fibronectin-coated glass bottom dishes (MatTek Corporation, Ashland, MA). Cells were cultured in reduced-serum media (MEM*α*+5% FBS+1% PenStrep) for 48 hours prior to fixation and received either 5 *μ*g/mL 4-hydroxytamoxifen (Sigma) dissolved in 90% ethanol or vehicle control for 24 hours prior to fixation. Cells were fixed in 10% formalin solution (Sigma), blocked in 10% goat serum (Thermo Fisher), incubated in a primary antibody against acetylated *α*-tubulin obtained from a C3B9 hybridoma (Sigma), and incubated in 1 : 500 Alexa Fluor 488 secondary antibody (Thermo Fisher). All blocking and antibody steps were for 1 hr at ambient temperature. Cell nuclei were stained with NucBlue solution (Thermo Fisher) for 5 minutes at ambient temperature. To quantify primary cilium length and incidence, a laser scanning confocal microscope (Leica TCS SP5, Leica Microsystems Inc., Buffalo Grove, IL) was used to collect at least 10 slices and create Z-stacks of cell nuclei and their associated cilia. These stacks were imported into ImageJ, and cilium length and incidence were measured manually by two separate investigators for accuracy and repeatability. Length was measured using a pixel to *μ*m conversion, and incidence was calculated using fields that were 80% confluent and contained 20-25 nuclei since primary cilium growth is influenced by cell density.

### 2.3. Mechanical Loading

One week prior to loading, peritoneal injections of 75 mg/kg body weight tamoxifen (1.5-2 mg dissolved in corn oil, Sigma) were administered once a day for 5 days to induce Cre recombination. Tamoxifen injections were also administered each day of loading for 3 days total and on days 4 and 8 after initiation of loading. At 16 ± 1 weeks of age, the right ulna of skeletally mature mice was exposed to compressive axial loading (3 N at 2 Hz for 120 cycles per day) for 3 consecutive days using an electromagnetic loading system (EnduraTEC, Bose, Eden Prairie, MN) [15, 21]. The left ulna was not loaded and served as an internal control. Mice revived from anesthesia within 5 minutes of completion of loading and normal gait were observed.

### 2.4. Dynamic Histomorphometry

Subcutaneous injections of calcein (30 mg/kg body weight, Sigma) and Alizarin Red S (70 mg/kg body weight, Sigma) were administered 4 and 8 days, respectively, after the first day of loading. Mice were sacrificed 14 days after initiation of loading, and ulnae were dissected, stored in 70% ethanol up to a week, dehydrated, and embedded in methyl methacrylate and dibutyl phthalate, using benzoyl peroxide as a catalyst. A diamond saw (IsoMet Low Speed Saw, Buehler, Lake Bluff, IL) was used to create transverse sections at the midshaft. Sections were imaged using a laser scanning confocal microscope (Leica TCS SP5, Leica Microsystems Inc., Buffalo Grove, IL). With ImageJ software, the following were measured for the periosteal surface: bone perimeter (B.Pm), single label perimeter (sL.Pm), double label perimeter (dL.Pm), and double label area (dL.Ar). These measures were used to calculate bone formation parameters: mineralizing surface (MS/BS = [1/2 sL.Pm + dL.Pm]/B.Pm × 100; %), mineral apposition rate (MAR = dL.Ar/dL.Pm/4 days; *μ*m per day), and bone formation rate (BFR/BS = MAR × MS/BS × 3.65; *μ*m^3^/*μ*m^2^ per day). Relative (*r*) measurements reflect bone formation that is attributed to mechanical loading and were calculated by subtracting nonloaded from the loaded forelimb values for individual animals.

### 2.5. Immunohistochemistry

Animals were injected and euthanized following loading as explained above. Both ulnae were dissected, fixed in 10% formalin (Sigma), decalcified in RDO, dehydrated, and embedded in paraffin. Transverse sections 5 *μ*m thick were cut at the midshaft. Sections were deparaffinized, rehydrated, blocked in 10% goat serum for 1 hr (Sigma), and then incubated with primary antibodies against GFP (1 : 500, Life Technologies, A11122) or beta-galactosidase overnight (1 : 3000, ab9361, abcam, Cambridge, UK). Sections were then incubated with biotinylated secondary antibodies for 30 min before adding ABC reagent (Vector Laboratories, Burlingame, CA) for 30 min. Development of color occurred through incubation in diaminobenzidine (DAKO, Carpinteria, CA) substrate solution. Finally, sections were counterstained with hematoxylin for 10 min. All incubations took place at ambient temperature.

### 2.6. Statistical Analysis

Data are presented as mean ± SEM and *p* values were calculated in GraphPad Prism (GraphPad Software, Inc., La Jolla, CA). One-way ANOVA revealed there were no statistical differences based on gender, so males and females were grouped together. Furthermore, one-way ANOVA demonstrated there was no difference in reported values due to the specific genotype for control animals with intact Kif3a. For dynamic histomorphometry comparisons, we could not assume normality (*n* < 30), so the nonparametric Mann-Whitney *U* test was used to identify any differences. Tests were performed with *α* = 0.05, and sample size was determined to achieve at least 80% power.

## 3. Results

### 3.1. Tamoxifen-Induced Cilium Disruption in Primary Periosteal Cells

The Prx1CreER-GFP; Kif3a^fl/fl^ and Prx1CreER-GFP genotypes were confirmed via standard PCR and gel electrophoresis (Figure 1(a)). We then isolated primary cells to evaluate our model's ability to disrupt primary cilia. Isolated Prx1CreER − GFP; Kif3a^fl/fl^ periosteal cells were identified via GFP expression *in vitro*. Indeed, Prx1-expressing cells treated with the active compound of tamoxifen had visually shorter cilia compared to vehicle controls (Figure 1(b)). More importantly, cilium incidence was 79 ± 1.4% (*n* = 4) in controls and 32 ± 1.4% (*n* = 5) in treated samples. Thus, we conclude that tamoxifen treatment effectively disrupts primary cilia in our transgenic mouse model.

### 3.2. Expression of Prx1 in Adults

**Figure 2:**
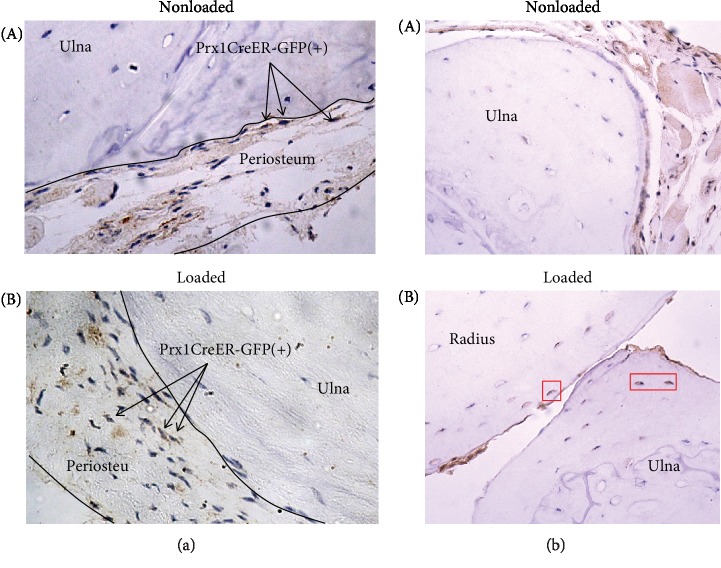
Prx1-expressing cells become embedded osteocytes in response to mechanical loading. (a) Prx1CreER-GFP(+) cells (brown) were detected in the periosteum surrounding the nonloaded (A) and loaded bone (B). Arrows denote examples of these cells for clarity. Osteocytes, muscle cells, bone marrow cells, and other cells in the periosteum were negative for GFP staining. (b) Beta-galactosidase staining was used to detect cells in which recombination had occurred (brown). Osteocytes near the periosteal surface demonstrated recombination in the loaded bone ((B), red boxes), but these cells were absent from nonloaded limbs (A). Brown coloration observed in the periosteum, muscle, and lacunae without corresponding nuclei (blue) was also seen in negative controls and thus deemed nonspecific staining. Nuclei were stained with hematoxylin, and micrographs were collected at 20x magnification.

Since there are conflicting reports on Prx1 expression in adults [4, 10–12, 22], we sought to determine whether cells containing the Prx1CreER-GFP transgene were present in the periosteum of skeletally mature mice. Immunohistochemistry was used to visualize GFP and therefore identify Prx1 expression (Figure 2(a)). We detected cells carrying the Prx1CreER-GFP transgene in the periosteum of loaded and nonloaded ulnae, indicating Prx1 is expressed under normal static conditions, as well as when mechanical loads are introduced. Not all cells in the periosteum expressed Prx1, which was absent in osteoblasts, osteocytes, bone marrow, and muscle surrounding the ulna.

### 3.3. Prx1-Expressing Cells Contribute to Load-Induced Bone Formation

We then tracked the fate of Prx1-expressing cells in the nonloaded and loaded ulna by staining for beta-galactosidase in our Prx1CreER-GFP; R26R reporter. We observed beta-galactosidase-positive cells within the periosteum of both loaded and nonloaded limbs (Figure 2(b)). Recombined cells were also uniquely present near the periosteal edge of the cortical bone in loaded limbs. Since osteocytes do not express the Prx1CreER-GFP transgene, this suggests that mechanical loading promotes osteogenic differentiation of Prx1-expressing progenitors.

### 3.4. Prx1-Driven Kif3a Deletion Diminishes Load-Induced Bone Formation

**Figure 3:**
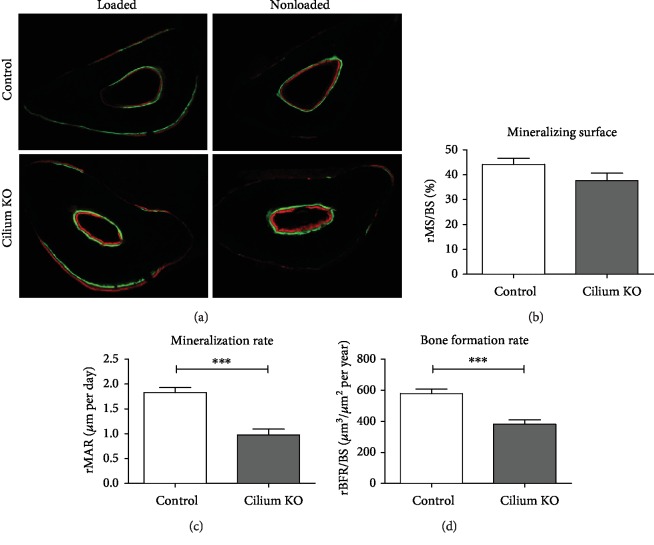
Load-induced bone formation is attenuated in mice with a Prx1-driven cilium knockout. Fluorochrome labels depicting load-induced bone formation at the periosteal surface (a). Dynamic histomorphometry to quantify bone formation parameters (c-d). Micrographs were collected at 10x magnification. *n* = 22 control and *n* = 28 experimental animals, ^∗∗∗^*p* < 0.001.

We then explored whether Prx1-expressing cells require primary cilia to contribute to adult bone formation. Mice with and without intact Prx1-expressing progenitor primary cilia were exposed to ulnar loading, and bone formation parameters were quantified using dynamic histomorphometry. Indeed, animals lacking cilia exhibited diminished bone formation (Figure 3(a)). Specifically, the relative bone formation rate (Figure 3(d)), which is a combination of mineralizing surface percentage and mineral apposition rate, was attenuated in experimental animals (381.7 ± 28.4 *μ*m^3^/*μ*m^2^/year) compared to controls (578.5 ± 28.4 *μ*m^3^/*μ*m^2^/year). The attenuated bone formation rate in mutants is due to a decrease in relative mineral apposition rate (1.0 ± 0.1 *μ*m/day compared to 1.8 ± 0.1 *μ*m/day for controls), which indicates how quickly new bone is formed at active surfaces (Figure 3(c)). Interestingly, we found no significant change (*p* = 0.11) in relative mineralizing surface (Figure 3(b)), or the percent of the surface that is active in bone formation, between animals with disrupted cilia (37.7 ± 3.0) compared to controls (44.2 ± 2.4%). Overall, our results indicate that Prx1-expressing progenitors require primary cilia to contribute to load-induced bone adaptation in adult mice.

## 4. Discussion

In this study, we identified Prx1-expressing cells as a source for osteoblasts active in load-induced bone formation. Osteoblast repopulation via progenitor cells has long been thought to drive adult bone formation, but no previous study has specifically traced the source of progenitors. Turner et al. used BrdU labeling to identify proliferating cells derived from progenitors after loading. On the endocortical surface, only 30-40% of osteoblasts were progenitor derived; however, on the periosteal surface, 90% of osteoblasts were newly created [23]. In a study of growing animals, bone formation was impaired when differentiated osteoblasts were ablated, while osteoprogenitors remained intact [24]. Once ablation ceased, bone formation returned to typical rates within 4 weeks, indicating that the osteoblast store had been repopulated from progenitor cells. These studies demonstrate that progenitors contribute to bone formation, but our work is the first to suggest active osteoblasts originate from Prx1-expressing progenitors. Specifically, our tracking studies reveal that some osteocytes near the periosteal surface arise from Prx1-expressing progenitors. A fraction of osteoblasts at the mineralizing surface eventually differentiate into osteocytes; therefore, the lacZ+ osteocytes we identified were once active osteoblasts.

Our results suggest that Prx1-expressing progenitors are recruited from the periosteum during load-induced bone formation. We identify four pieces of evidence that support this theory. First, our histological analysis and preexisting literature suggest Prx1 expression is confined to the periosteum in adult mice. Various tracking studies in constitutive Prx1Cre models report labeled cells in a variety of tissues [9–11], but these studies do not examine Prx1 expression specifically in adulthood. Kawanami et al. first reported that Prx1 expression becomes highly restricted after birth [4]. Duchamp De Lageneste et al. demonstrated that rare periosteal Prx1-expressing cells persist in the periosteum after healing is complete [14]. In our prior research, we detected Prx1 expression using a fluorescent reporter and concluded it was highly restricted to the periosteum with age [12, 13]. Here, we stained for GFP in skeletally mature adults and determined the Prx1CreER-GFP transgene was present in the periosteum in loaded and nonloaded bone, but not found in osteoblasts, osteocytes, muscle, or bone marrow. It is important to note that we only evaluated tissues spatially relevant to the ulna, so Prx1 may be expressed elsewhere. For example, Prx1 expression is also present in the calvaria after birth [25], but we do not anticipate that the expression in the areas of the body outside the area we evaluated would influence our findings.

Second, Kawanami et al. found that primary Prx1-expressing cells isolated from long bones of Prx1CreER-GFP mice were periosteal in nature [4]. Specifically, Prx1-expressing cells were sorted using the GFP tag, and quantitative PCR was performed to measure typical cell markers associated with various skeletal cells. The expression patterns of the primary cells were by far most consistent with periosteal cells, especially when compared to the expression patterns of osteoblast, preadipocyte, and macrophage cell lines.

Third, since bone formation primarily occurs at the periosteal surface in mice, it is widely believed that active osteoblasts are recruited from the periosteum, which is intimately connected to the periosteal bone surface [23, 26]. In fact, the recombined osteocytes we found in our tracking studies were all located near the periosteal surface. This spatial positioning strongly suggests active osteoblasts are derived from Prx1-expressing progenitors in the periosteum. Furthermore, in the tracking studies of their Prx1CreER-GFP model, Kawanami et al. noted that recombined cells were predominantly in the inner cambium layer [4], which is connected to the bone surface and readily provides osteoprogenitors [27].

Fourth, our dynamic histomorphometry data suggest Prx1-expressing progenitors are not derived from bone marrow, which is another expected source. We previously demonstrated that marrow-derived MSCs require primary cilia to populate the cortical bone at the periosteal surface in response to mechanical loading [28]. This suggests that Prx1-expressing progenitors containing disrupted cilia in the marrow would not mobilize to the periosteal surface. However, we found that the mineralizing surface is unchanged in mutants, meaning active osteoblasts still arrive at the periosteal surface. These phenomena are therefore inconsistent, and our data further implicate the periosteum as the source of Prx1-expressing progenitors. Additionally, we did not detect Prx1 expression in bone marrow when we visualized GFP. Collectively, our results provide compelling evidence to suggest Prx1-expressing progenitors that participate in load-induced bone formation are recruited from the periosteum.

The bone-forming activity of osteoblasts derived from Prx1-expressing progenitors is dependent on primary cilia, but recruitment and differentiation occur independently. The relative percent mineralizing surfaces (rMS/BS) were equivalent between experimental and control animals. This is surprising since the majority of active osteoblasts are derived from recruited progenitors at the time points we evaluated [23] and therefore suggests that primary cilia do not influence progenitor recruitment in this instance. We also found that the relative rate of mineral apposition (rMAR) of mutant animals was approximately half the rate of controls, indicating primary cilia play a potent role in osteoblast activity. In a previous study by Temiyasathit et al., deletion of Kif3a in osteoblasts and osteocytes also resulted in a decrease in rMAR (~30%), with no change in rMS/BS [18]. The greater decrease in rMAR in our current study may be due to the deletion occurring prior to osteoblast differentiation. Mutants maintain some mineral apposition, suggesting that other cell types also contribute to adaptation. For example, the earliest active osteoblasts in response to loading are likely derived from bone lining cells [23]. Despite these alternative contributions, the severity of attenuated bone formation in mutants suggests Prx1-expressing cells have a significant impact.

**Figure 4:**
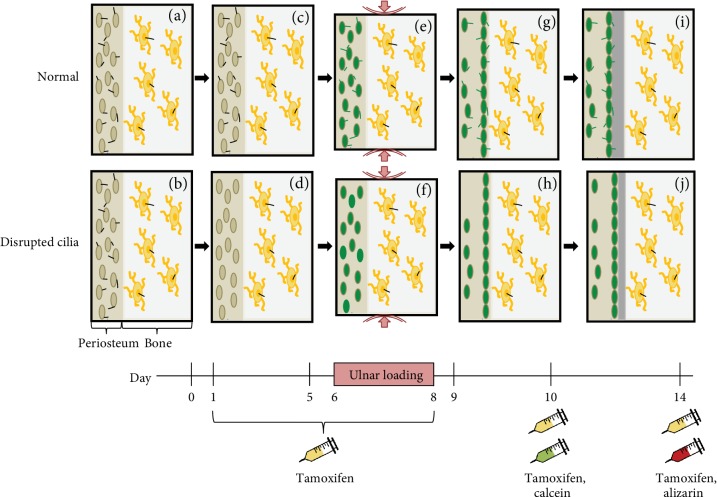
Proposed effect of Prx1-driven primary cilium disruption on load-induced bone formation. Prx1-expressing periosteal progenitor cells (tan) and osteocytes (yellow) contain functional primary cilia (black) prior to experimentation (a, b). Tamoxifen is injected prior to loading, and periosteal progenitor primary cilia are uniquely disrupted in mutant mice (c, d). Ulnar loading is applied and both periosteal progenitors and osteocytes sense the mechanical stimulus. Periosteal progenitors are activated (green) by loading, regardless of whether their primary cilia are functional (e) or disrupted (f). The majority of activated progenitors migrate to the cortical surface (g, h). A continuous layer of matrix is deposited on the periosteal surface, but periosteal progenitors with disrupted cilia (j) produce less newly formed bone than those with functional cilia (i).

Although our data indicate Prx1-expressing cells contribute to mechanically induced bone formation in a primary cilium-mediated process, the exact mechanism is unknown. The primary cilium is both a mechano- and chemosensor, so disrupting this organelle potentially abrogates a progenitor cell's ability to detect both physical and biochemical stimuli. One intriguing possibility is that mechanical stimulation alone directs progenitors to embark on a preprogrammed path of becoming active osteoblasts. Without functional primary cilia, Prx1-expressing progenitors are not properly encoded to form bone once they arrive at the bone surface (Figure 4). This situation may not be entirely novel, since bone loss due to disuse is a consequence of decreased bone formation by osteoblasts in the basic multicellular unit [29]. Another possibility is that osteocytes sense loading and signal to Prx1-expressing cells, triggering differentiation. In this scenario, Prx1-expressing cells without primary cilia would exhibit dysfunctional chemosensation and fail to properly transduce signals from mechanically stimulated osteocytes. These two phenomena are not mutually exclusive so a third speculation is that Prx1-expressing progenitors both directly sense mechanical stimulation and receive signals from osteocytes.

One limitation of this study is that Kif3a has been linked to nonciliary functions. Loss of Kif3a results in constitutive phosphorylation of Dishevelled, leading to overactivation of the canonical Wnt pathway [30], which is believed to mediate progenitor differentiation [31, 32]. However, deletion of Ift88, another gene that affects ciliogenesis, did not cause constitutive phosphorylation of Dishevelled. Deletion of Ift88 may therefore be a more specific model for disrupting primary cilia, but Prx1-driven *in vivo* deletion of Kif3a and Ift88 in embryonic studies produced identical results [16]. Regardless, the results presented here are novel in showing that disruption of a key ciliary protein in a progenitor source causes a deficiency in load-induced bone formation.

## 5. Conclusions

In this study, we demonstrate for the first time that Prx1-expressing cells contribute to mechanically induced bone formation. We also found evidence that, while functional primary cilia are not necessary for these progenitors to arrive at the bone surface, they are important for bone apposition activity once these cells differentiate into osteoblasts. The primary cilium may serve as a microdomain that facilitates signaling by enhancing reaction kinetics or bringing specific reaction partners together. Although this organelle's role in bone apposition remains unknown, understanding activation of periosteal progenitors through primary cilium-mediated mechanisms would greatly focus the search for bone regeneration strategies.

## Data Availability

Primer sequences and data used to support the findings of this study are available from the corresponding author upon request.
